# Community and Health Care Provider Preferences for Bacterial Sexually Transmitted Infection Testing Interventions for Gay, Bisexual, and Other Men Who Have Sex With Men: e-Delphi Study

**DOI:** 10.2196/40477

**Published:** 2023-06-29

**Authors:** Anna Yeung, Ryan Lisk, Jayoti Rana, Charlie B Guiang, Jean Bacon, Jason Brunetta, Mark Gilbert, Dionne Gesink, Ramandip Grewal, Michael Kwag, Carmen H Logie, Leo Mitterni, Rita Shahin, Darrell HS Tan, Ann N Burchell

**Affiliations:** 1 MAP Centre for Urban Health Solutions Unity Health Toronto Toronto, ON Canada; 2 ACT Toronto, ON Canada; 3 Department of Family and Community Medicine University of Toronto Toronto, ON Canada; 4 Ontario HIV Treatment Network Toronto, ON Canada; 5 Maple Leaf Medical Clinic Toronto, ON Canada; 6 British Columbia Centre for Disease Control Vancouver, BC Canada; 7 Dalla Lana School of Public Health University of Toronto Toronto, ON Canada; 8 Community-Based Research Centre Vancouver, BC Canada; 9 Factor-Inwentash Faculty of Social Work University of Toronto Toronto, ON Canada; 10 Women’s College Research Institute Women’s College Hospital Toronto, ON Canada; 11 Centre for Gender & Sexual Health Equity Vancouver, BC Canada; 12 Hassle Free Clinic Toronto, ON Canada; 13 Toronto Public Health Toronto, ON Canada

**Keywords:** sexual and gender minorities, sexually transmitted diseases, community-based research, mass screening, patient acceptance of health care

## Abstract

**Background:**

Canadian clinical guidelines recommend at least annual and up to quarterly bacterial sexually transmitted infection (STI) testing among sexually active gay, bisexual, and other men who have sex with men (GBM). However, testing rates are suboptimal. Innovative solutions are needed to close the gap because there is currently limited knowledge on how best to approach this issue.

**Objective:**

Our aim was to build consensus regarding interventions with the greatest potential for improving local STI testing services for GBM communities in Toronto, Ontario, Canada, using a web-based *e-Delphi* process.

**Methods:**

The e-Delphi method involves using a panel format to conduct successive rounds of prioritization, with feedback between rounds, to determine priorities among groups. We recruited experts separately from the community (GBM who sought or underwent STI testing in the preceding 18 months; conducted between October 2019 and November 2019) and health care providers (those who offered STI testing to GBM in the past 12 months; conducted between February 2020 and May 2020). The experts prioritized 6 to 8 potential interventions on a 7-point Likert scale ranging from *definitely not a priority* to *definitely a priority* over 3 survey rounds and ranked their top 3 interventions. Consensus was defined as ≥60% within a ±1 response point. Summaries of responses were provided in successive rounds. We reported the percentage of *a priority* (encompassing *somewhat a priority*, *a priority,* and *definitely a priority* responses) at the end of the final round of the survey.

**Results:**

Of the community experts (CEs), 84% (43/51) completed all rounds; 19% (8/43) were living with HIV; 37% (16/43) were HIV negative and on pre-exposure prophylaxis; and 42% (18/43) were HIV negative and not on pre-exposure prophylaxis. We reached consensus on 6 interventions: client reminders (41/43, 95%), express testing (38/43, 88%), routine testing (36/43, 84%), an online booking app (36/43, 84%), online-based testing (33/43, 77%), and nurse-led testing (31/43, 72%). The CEs favored convenient interventions that also maintain a relationship with their provider. Of the provider experts (PEs), 77% (37/48) completed all rounds; 59% (22/37) were physicians. Consensus was reached on the same 6 interventions (range 25/37, 68%, to 39/39, 100%) but not for provider alerts (7/37, 19%) and provider audit and feedback (6/37, 16%). Express testing, online-based testing, and nurse-led testing were prioritized by >95% (>37/39) of the PEs by the end of round 2 because of streamlined processes and decreased need to see a provider.

**Conclusions:**

Both panels were enthusiastic about innovations that make STI testing more efficient, with express testing rating highly in both the prioritizations and top 3 rankings. However, CEs preferred convenient interventions that involved their provider, whereas PEs favored interventions that prioritized patient independence and reduced patient-provider time.

**International Registered Report Identifier (IRRID):**

RR2-10.2196/13801

## Introduction

### Background

In Canada, rates of bacterial sexually transmitted infections (STIs)—syphilis, gonorrhea, and chlamydia—have risen dramatically since 2000 [1], particularly among gay, bisexual, and other men who have sex with men (GBM). STI testing and treatment mitigate adverse health outcomes and substantially reduce transmission, yet testing rates remain below recommended levels based on patterns previously seen in GBM living with HIV [2,3]. Within the province of Ontario, from 2011 to 2019, cases of chlamydia, gonorrhea, and syphilis in men increased by 36%, 67%, and 63%, respectively [4-6]. The highest number of cases were reported in Toronto, the largest city in Ontario; in 2019, chlamydia, gonorrhea, and syphilis case counts were 34%, 49%, and 59% of the provincial total, respectively. These are likely undercounts owing to the asymptomatic nature of these infections. This situation is similar to that in many urban settings in high-income countries such as the United States [7], the United Kingdom [8], and Australia [9]. The recent COVID-19 pandemic also disrupted many sexual health services around the world, leading to an unmet need for STI testing [10,11]. Left untreated, bacterial STIs can result in sequelae such as epididymo-orchitis [12] and infertility [13] from chlamydia and multidrug antibiotic resistance [14] for gonorrhea treatment, as well as systemic spread to other organs in the case of syphilis [15]. All bacterial STIs may increase the risk of HIV acquisition, transmission, and viral load [16].

Without increasing STI awareness and prevention through the promotion of safer sex practices or expanding access to testing services, advances in HIV medicine such as pre-exposure prophylaxis (PrEP) and treatment as prevention may unintentionally fuel bacterial STI transmission through a potential decline in the use of condoms [17]. The increase in STIs globally needs local innovation to produce the required uptake of testing, including increasing the test coverage, frequency, and use of appropriate testing technologies, in ways that are engaging, nonstigmatizing, and acceptable. One strategy to mitigate the rise in STIs is the *test and treat* strategy where individuals are tested and treated to shorten the duration of infectiousness, thus reducing transmission and adverse health outcomes [18,19]. However, it is not clear how to optimally implement this model among GBM in the Toronto setting.

Canadian STI guidelines recommend annual screening among sexually active GBM and more frequently for individuals of all genders based on behavioral risk factors [20]*.* Recent data from the Engage study of GBM living in Toronto, Montreal, and Vancouver showed that 67% to 79% of GBM had been tested in the previous 6 months, but 13% to 18% had never been tested [21]. GBM from the Engage study were also tested at baseline and showed a prevalence ranging from 3% to 6% for chlamydia, 2% to 9% for gonorrhea, and 14% to 16% for a history of syphilis infection. Fewer GBM undergo recommended extragenital testing, with 32% to 65% testing for rectal and pharyngeal gonorrhea and chlamydia compared with 82% for urogenital gonorrhea and chlamydia [22], which could miss between 71% and 100% of infections [23,24]. Focus groups of GBM conducted previously by our team identified barriers to testing in Toronto [25], including the lack of accessibility to sexual health clinics because of geographic location, limited opening hours, language and other cultural barriers, and long wait times. In addition, issues with delivery of bacterial STI testing were raised, including providing a sexual history, knowledge of what tests are needed, standardizing follow-up results, and public health reporting for men living with HIV.

### Objective

Evidence is needed for locally informed solutions to improve access to, and uptake of, bacterial STI testing. Our objective was to build consensus regarding interventions with the greatest potential for improving local STI testing services for GBM communities in Toronto using a web-based *e-Delphi* process.

## Methods

### e-Delphi Study Design

We conducted two separate Delphi studies for (1) provider experts (PEs): health care providers and public health professionals with expertise in providing STI testing for GBM communities and (2) community experts (CEs): members of the GBM community with lived experience who have sought testing. The protocol for this study has been published [26]. Here, we report its results using the Conducting and Reporting of Delphi Studies standard [27]. This study was led by a team of experienced researchers, including members from the GBM community and health care providers.

Delphi studies are consensus-building exercises, characterized by four methodological features: (1) a group of experts are surveyed about an issue of interest, (2) the process is kept anonymous to avoid social pressure and conformity, (3) an iterative approach that comprises several rounds of inquiry is used, and (4) the design of subsequent rounds is informed by a summary of the group response of the previous round [27]. This iterative process makes it ideal for use within community-based and patient-oriented research and can be adapted for a web-based format (*e-Delphi*). The Delphi method allows community members to have an equal voice alongside health care providers and has merit as a tool to improve the quality of health care services [28]. It also allows participants to express opinions first without influence and then adjust opinions with feedback from their peers.

Our e-Delphi study comprised two phases: (1) identifying interventions to include in the e-Delphi survey for voting [26] and (2) the e-Delphi process itself. The second phase is the focus of this paper. During the first phase, interventions to include in the e-Delphi study were identified using a 3-step process. First, we conducted a review of the published literature to select STI testing interventions. The publications included were subdivided into 3 major categories as defined in our protocol: interventions to streamline testing among asymptomatic individuals, client-targeted testing interventions, and provider-targeted testing interventions [26]. These 3 intervention groupings had moderate or high effectiveness in previous studies [29]. Next, focus groups of GBM, including those who were HIV positive, those who were HIV negative, and those who identified as transgender, were held to identify barriers and facilitators to bacterial STI testing to further refine the interventions [25]. The men identified that optimal testing is enabled by variety and choice in testing options, delivered as person-centered, lesbian, gay, bisexual, and transgender–affirming care. Finally, health care providers based in primary care and sexual health settings were surveyed about their current practices as well as barriers and attitudes to improve testing rates [30]. The providers favored initiatives that addressed barriers of insufficient consultation time, capacity, and resources by simplifying and expediting procedures.

**Figure 1:**
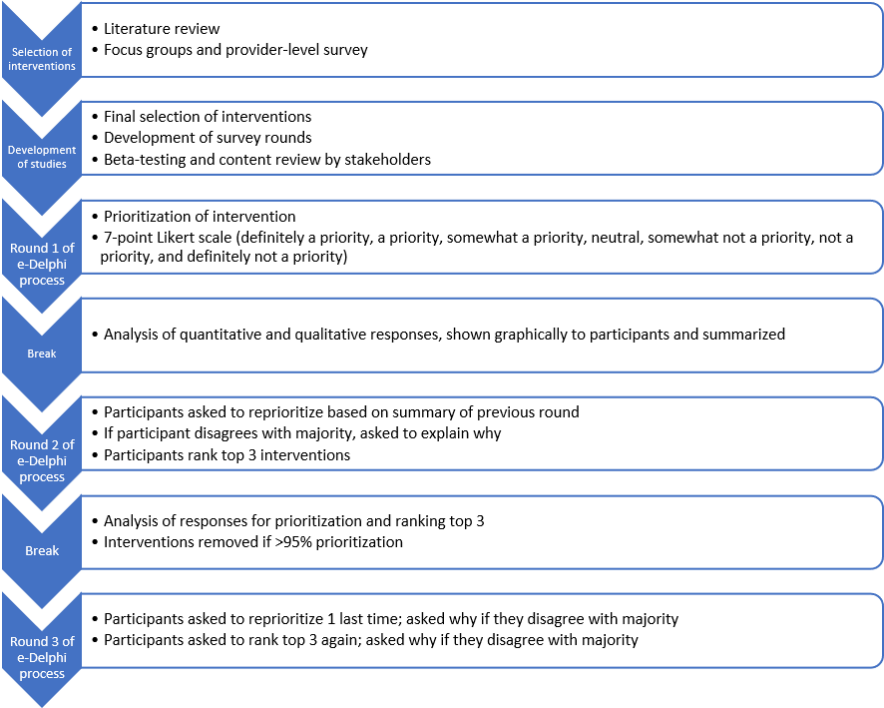
Development and process of the Delphi study.

After refining the list of potential interventions based on our literature review, focus groups, and the provider-level survey, we used a modified web-based e-Delphi study to build consensus on which the interventions would be prioritized by stakeholders (Figure 1). We chose the Delphi method to identify which intervention would be best suited for implementation in the sexually, racially, and ethnically diverse Toronto setting.

At the end of the first phase, the final interventions were grouped into 3 major categories (Table 1): streamlining STI testing for asymptomatic individuals, client-targeted interventions, and provider-targeted interventions. CEs saw the first 2 categories, which comprised 6 interventions in total. PEs saw all 3 categories, which comprised 8 interventions in total.

**Table 1:** Description of interventions presented to participants.

Category and intervention name		Description of intervention
**Streamlining STI^a^ testing for asymptomatic individuals**		
	Online-based bacterial STI testing	For online-based bacterial STI testing, the patient would request the test from a website, download a laboratory form, and visit a laboratory in person to provide samples (eg, anal swab, throat swab, or urine samples for chlamydia and gonorrhea and blood sample for syphilis).
	Express testing at clinics with self-collection of samples	Patient goes to a clinic and fills out symptoms and sexual history questionnaire. If symptomatic, the patient sees a health care provider and proceeds with a clinical examination. If asymptomatic, the questionnaire recommends which samples should be self-collected for testing. To self-collect samples, patients will be provided with a private space with posters describing how to collect each sample. Samples that could be self-collected include urine, rectal, or throat swab. Self-collected samples are placed in a bin for processing. There is no clinical examination.
	Nurse-led testing in primary care clinics	Patient attends a primary care clinic and is screened by a nurse or allied health professional who asks questions about STI symptoms and sexual history using standardized questions. If the patient has symptoms, they will see the physician^b^. If the patient does not have symptoms, their sexual history helps to determine which tests should be offered. Patients are called back if the results of the test are positive and treatment is required.
	Routine testing	STI testing is routinized using standing orders: tests that are provided at health care visits for other purposes, such as annual visits and as part of HIV care (if they are living with HIV), or when they see their physician for another health issue, such as strep throat or pain in their shoulder.
**Client-targeted interventions**		
	Client reminders	Patients who have had STI tests before and have consented to being contacted between visits would receive reminders to return for their next STI test via SMS text message, email, or mail. The reminder message would look similar to the following template: “Hi [patient name], it’s time for a routine test. Walk in, call [xxxxxx], or email [xxxxxxxx] for an appointment.”
	Online booking app for STI testing	Patient uses an app or visits a website that has information about bacterial STIs, such as the ways in which these infections can be transmitted, the symptoms of infection, and how the tests are conducted. Patient can use the website or app to book an appointment for an STI test at a clinic near them.
**Provider-targeted interventions**		
	Provider alerts	Health care providers receive alerts through EMR^c^ systems to prompt an offer of STI testing. Alerts could be triggered when clients are due for STI retesting or if a client self-completes a sexual history questionnaire and reports high-risk sexual activity.
	Provider audit and feedback	A person or organization has access to health care provider–level STI testing data and prepares regular reports. Health care providers receive regular customized reports about their own STI testing practice patterns and how they compare with guideline recommendations, their own historical STI testing pattern, and that of other providers. The report also contains actionable goals to achieve STI testing targets, encouraging improvement and reinforcing good performance. It also promotes healthy competition by showing providers how they are doing compared with their peers.

^a^STI: sexually transmitted infection.

^b^“Doctor” instead of “physician” was shown to participants for accessibility reasons.

^c^EMR: electronic medical record.

### Development of Data Collection Instruments for the e-Delphi Survey Rounds

We prepared short summaries of each intervention that described which actions would or would not be involved, tailored to the CEs and PEs (Table 1; Multimedia Appendix 1). Preliminary versions of the survey questionnaires were then pilot-tested. For the CE surveys, members of our community partner organization, ACT (formerly the AIDS Committee of Toronto), provided feedback on language, flow, and appropriateness. For the PE surveys, health care providers on the study team reviewed the questionnaires to ensure content relevance, appropriate language, and comprehensibility. All data collection instruments were developed and delivered using Qualtrics, a web-based survey platform. Data collected via Qualtrics were stored on servers in Canada and protected with high-end firewalls.

### Ethics Approval

Ethics approvals were obtained from the research ethics boards at the University of Toronto (37620) and Unity Health Toronto (17-176).

### Participant Recruitment

We recruited CEs using several strategies over a 2-week period. A poster was produced and shared in local gay, bisexual, and queer community spaces, including a bookshop specializing in queer literature and posting boards of community organizations, whereas an electronic version was shared on the social media accounts, including Facebook, Instagram, and Twitter, of our community partner organization. We sent recruitment emails to HIV and AIDS service organizations that served specific ethnoracial groups. In addition, advertisements ran on Grindr, a gay dating app. For the CEs, we aimed to have 40% of the participants with non-White ethnoracial identity and 40% aged <40 years to represent the racial diversity of the GBM community in Toronto. Eligible CEs (1) were aged ≥18 years; (2) lived in Toronto; (3) identified as men; (4) were sexually active with another man in the past 18 months; and (5) reported that they tried to get or had gotten an STI test for chlamydia, gonorrhea, or syphilis in the past 18 months. Respondents completing the eligibility survey were asked to provide the first 3 digits of their postal code to verify that they lived in Toronto.

Members of our team suggested potential PEs based on our professional networks. We then extended invitations for participation using direct emails over a 3-week period. All PEs were directed to complete a survey to determine eligibility. PEs had to (1) have the majority of their clinical practice based in Toronto, (2) see ≥1 GBM per week in the clinic, (3) be involved in some aspect of STI testing or management, and (4) provide STI tests to GBM ≥1 times per month.

After completing the eligibility survey, participants provided written informed consent to participate in either the CE or PE studies, as appropriate. We aimed to recruit 45 respondents per study to ensure that at least 30 completed all 3 rounds.

### Data Collection

Participants were sent links to complete the e-Delphi survey rounds either by email or SMS text message. Participants were compensated for their time for each round of the survey: CAD $25 (US $18.8) for round 1, CAD $35 (US $26.3) for round 2, and CAD $40 (US $30.0) for round 3, for a possible total of CAD $100 (US $75.1). Those who completed a round were invited back to subsequent rounds. CEs were given 2 weeks to complete each survey round, whereas PEs were given 3 weeks. There was a 1-week break between rounds. Multiple reminders were sent during rounds to maximize completion.

Each survey round asked participants to rate interventions on a 7-point Likert scale: definitely a priority, a priority, somewhat a priority, neutral, somewhat not a priority, not a priority, or definitely not a priority. In addition, in rounds 2 and 3, we asked participants to rank their top 3 interventions. Open-text questions were asked regarding the need for additional information or context to make a decision, and space was provided to explain their ratings. Subsequent rounds showed the distribution of responses, and participants were asked to reprioritize. If they disagreed with the majority, they were provided space to explain their disagreement. Participants also provided sociodemographic information at the end of each round.

The PE study was interrupted by the onset of the COVID-19 pandemic in Toronto, with the provincial government declaring a state of emergency on March 17, 2020. Round 1 of the study took place in the second half of February 2020. We delayed round 2 for PEs by 1 week to adjust for working conditions during the pandemic. However, we did not modify questionnaire items to ask about specific impacts of the COVID-19 pandemic.

### Data Analysis

We included a participant’s survey questionnaire data for a given round only if they completed that questionnaire; incomplete surveys were removed from the final data set.

We analyzed intervention prioritizations using the 7-point Likert scale with frequency counts. We also collapsed the 7-point responses into three main categories: (1) not a priority (*definitely not a priority*, *not a priority*, and *somewhat not a priority*), (2) neutral, and (3) a priority (*somewhat a priority*, *a priority*, and *definitely a priority*). We defined consensus as having ≥60% of the experts indicate a preference within 2 adjacent response points (±1); this threshold was decided in a discussion among our team members owing to the lack of a uniform definition for consensus in Delphi studies [31,32]. Open-text responses were thematically coded and summarized for participants in rounds 2 and 3. Top 3 rankings were counted by the number of votes in rounds 2 and 3; we reported the top 3 rankings back to participants in round 3.

## Results

### CE Study

The CE study was conducted from October 4, 2019, to November 30, 2019, and included 51 participants, of whom 46 (90%) completed the first round of the survey. Few participants were lost to follow-up, with 96% (44/46) of the participants completing round 2 and 98% (43/44) completing round 3. The majority of the CEs (35/46, 76%) were aged <40 years, and most of them (40/46, 87%) identified as gay (Table 2). Similar to the ethnoracial population of Toronto [33], a little more than half (24/46, 52%) of the participants identified as part of an ethnoracial group, such as African, Caribbean, and Black (3/46, 7%) or Middle Eastern (4/46, 9%; Table 2). One-fifth (9/46, 20%) of the participants were living with HIV, and the remainder were HIV negative (37/46, 80%); nearly half (18/37, 49%) of these participants were on PrEP.

**Table 2:** Demographics of community experts.

			Round 1 (n=46), n (%)	Round 2 (n=44), n (%)	Round 3 (n=43), n (%)
**Age (years)**					
	18-29	16 (35)		16 (36)	16 (37)
	30-39	19 (41)		18 (41)	17 (40)
	40-49	7 (15)		6 (14)	6 (14)
	≥50	4 (9)		4 (9)	4 (9)
**Identity^a^**					
	Gay	40 (87)		39 (89)	38 (88)
	Bisexual	2 (4)		2 (5)	2 (5)
	Queer	4 (9)		3 (7)	3 (7)
**Race, ethnicity, or heritage**					
	African, Caribbean, and Black	3 (7)		3 (7)	3 (7)
	Asian or Pacific Islander	8 (17)		7 (16)	7 (16)
	Indigenous	0 (0)		0 (0)	0 (0)
	Latinx and Hispanic	3 (7)		3 (7)	3 (7)
	Middle Eastern	4 (9)		4 (9)	4 (9)
	South Asian	2 (4)		2 (5)	2 (5)
	White	22 (48)		20 (45)	19 (44)
	Another identity^b^	4 (9)		5 (11)	5 (12)
**HIV serostatus**					
	HIV-positive	9 (20)		8 (18)	8 (19)
	HIV-negative, on PrEP^c^	18 (39)		16 (36)	16 (37)
	HIV-negative, not on PrEP	19 (41)		18 (41)	18 (42)
	Prefer not to answer	0 (0)		2 (5)	0 (0)
	I don’t know	0 (0)		0 (0)	1 (2)

^a^Participants were asked whether they identified as one of the following: gay, bisexual, queer, or 2-spirit or whether they preferred to use another term. None identified as 2-spirit or used another term.

^b^Responses included mixed race, Métis, Jewish, and Acadian.

^c^PrEP: pre-exposure prophylaxis.

### PE Survey

The PEs were surveyed from February 21, 2020, to May 13, 2020, and included 48 providers, of whom 45 (94%) completed round 1 of the survey. Few participants were lost to follow-up, with 87% (39/45) completing round 2 and 95% (37/39) completing round 3. Most of the PEs were aged <40 years (30/45, 67%), identified as men (26/45, 58%), practiced in primary care (25/45, 56%), had graduated <10 years ago (27/45, 60%), and were physicians (25/45, 56%; Table 3).

Participants were experienced in sexual health care, with the majority reporting seeing ≥20 GBM patients per week (23/45, 51%) and ≥20 bacterial STI tests ordered per month (28/45, 62%; Table 3). From round 1 to round 3, we saw small decreases in the number of woman providers (from 18/45, 40%, to 13/37, 35%), the number of providers who had graduated 10 to 19 years ago (from 11/45, 24%, to 6/37, 16%), and the number of providers who were seeing 10 to 20 GBM in practice per week (from 13/45, 29%, to 6/37, 16%). Unanimous consensus was reached for express testing, online-based testing, and nurse-led testing at the end of round 2, and PEs were not asked to reprioritize these in round 3.

**Table 3:** Demographics of provider experts.

			Round 1 (n=45), n (%)		Round 2 (n=39), n (%)		Round 3 (n=37), n (%)
**Age (years)**							
	<40	30 (67)		27 (69)		27 (73)	
	40-49	10 (22)		7 (18)		6 (16)	
	≥50	5 (11)		5 (13)		4 (11)	
**Gender identity**							
	Woman	18 (40)		14 (36)		13 (35)	
	Man	26 (58)		24 (62)		23 (62)	
	Self-described^a^	1 (2)		1 (3)		1 (3)	
**Practice setting**							
	Primary care	25 (56)		24 (62)		22 (59)	
	Sexual health	12 (27)		7 (18)		9 (24)	
	Other^b^	8 (18)		8 (21)		6 (16)	
**Years since graduation**							
	Not yet graduated	1 (2)		1 (3)		1 (3)	
	<10	27 (60)		25 (64)		25 (68)	
	10-19	11 (24)		7 (18)		6 (16)	
	≥20	6 (13)		6 (15)		5 (14)	
**Type of health care provider**							
	Counselor	1 (2)		1 (2)		1 (3)	
	Other^c^	1 (2)		1 (2)		0 (0)	
	Physician	25 (56)		25 (56)		22 (59)	
	Registered nurse	18 (40)		18 (40)		14 (38)	
**Number of GBM^d^ seen in practice per week**							
	1-9	9 (20)		5 (13)		10 (27)	
	10-19	13 (29)		11 (28)		6 (16)	
	≥20	23 (51)		23 (59)		21 (57)	
**Number of bacterial STI^e,f^ tests ordered per month**							
	1-9	8 (18)		3 (8)		5 (14)	
	10-19	9 (20)		8 (21)		6 (16)	
	≥20	28 (62)		28 (72)		26 (70)	

^a^Identified as nonbinary.

^b^Public health units, research, sexually transmitted infections case management, and both sexual health and family medicine.

^c^Public health nurse.

^d^GBM: gay, bisexual, and other men who have sex with men.

^e^STI: sexually transmitted infection.

^f^Chlamydia, gonorrhea, and syphilis

### Online-Based Bacterial STI Testing

**Figure 2:**
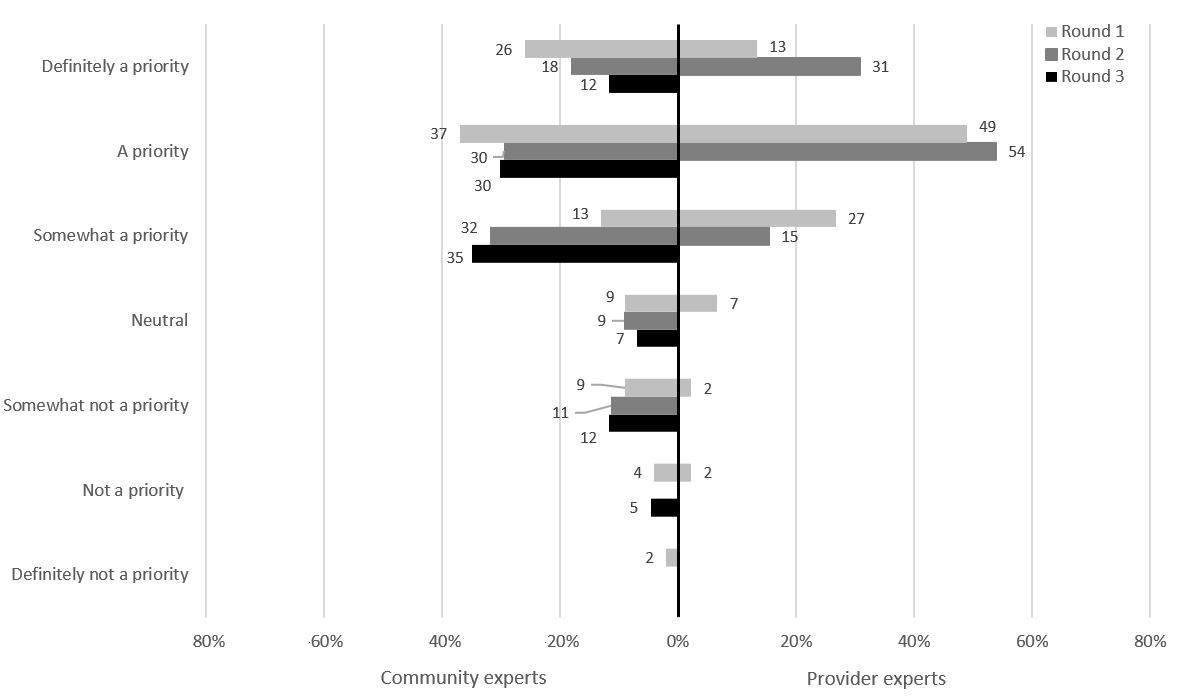
Online-based bacterial sexually transmitted infection testing: prioritization by participants.

In the first potential intervention presented to participants, online-based testing would allow participants to bypass visiting a health care provider and head directly to the pathology laboratory to complete STI testing. Figure 2 shows the proportion of prioritization by the experts in each round for online-based STI testing. For 77% (33/43) of the CEs, online-based STI testing was a priority at the end of round 3. Of the PEs, 100% (39/39) rated online-based STI testing as a priority at the end of round 2; because unanimous consensus was reached early, this intervention was not presented to PEs in round 3.

CEs and PEs had similar comments to justify their ratings. Many of them thought that online-based testing would increase accessibility and availability of testing, particularly for those who had not disclosed their sexual identity, did not have access to culturally sensitive care, or had a fear of being judged on their sexual practices. For such individuals, the participants felt that going directly to the laboratory for testing was less conspicuous and would reduce the shame and stigma associated with STI testing. However, a few of the participants raised the point that accessibility would be restricted to those with access to the internet and may not reach those at higher risk for STIs. Some of the participants also indicated that an opportunity for in-person counseling would be lost, and some brought up concerns around logistics, such as follow-up on positive results and fears of collecting specimens inaccurately. Both groups identified that leveraging technology would make testing more convenient, easier, and quicker, as long as the technology was secure and confidential:

This would make it a lot easier and quicker to get routine testing completed. The clinic I usually go to can have a 1-2 hour wait. By having an online test, it would shorten the wait times and let the guys that are seeking help with a symptom not have to wait hours to be seen.

CE 005

Cost was another theme brought up by both groups but for different reasons. CEs were less keen on a service if there were expenses involved. Some of the PEs expressed concerns regarding overtesting and increased costs to the health care system, whereas others thought that online-based testing would increase efficiency in testing:

It is important to build this system out and put it in place, as for some patients, this will improve access to testing services. This also has the potential to offload significant burden from existing clinics providing sexual health services (such as mine), saving the system time and dollars.

PE 031

### Express Testing at Clinics With Self-Collection of Samples

**Figure 3:**
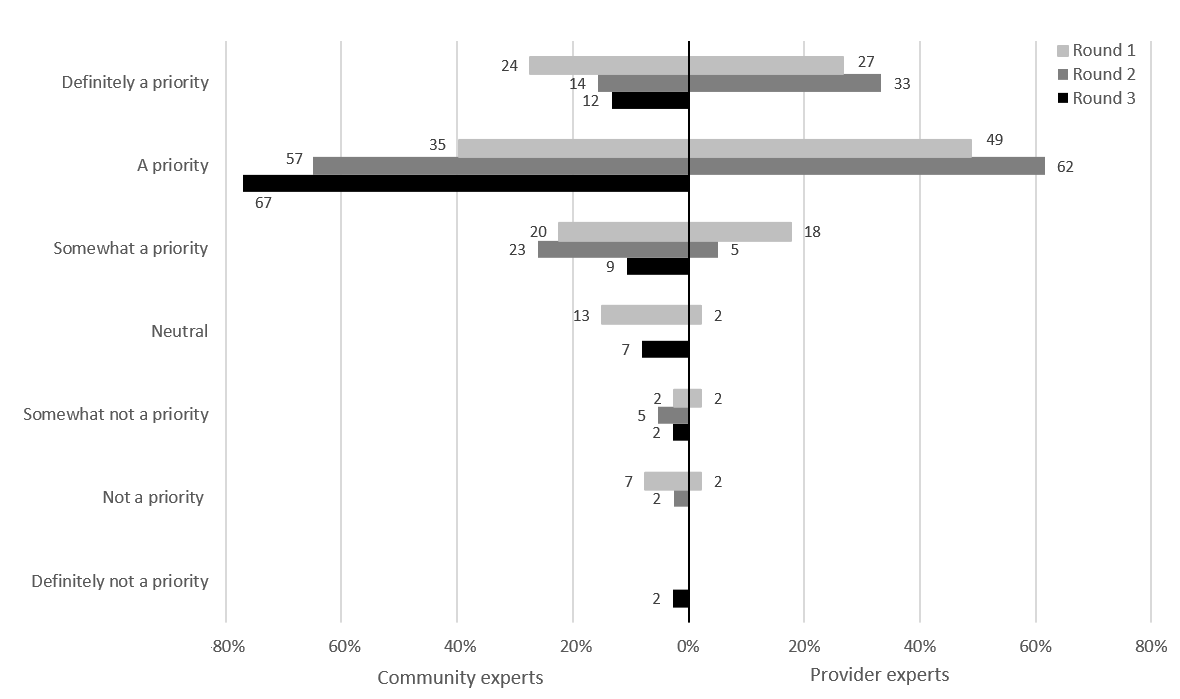
Express testing: prioritization by participants.

For this potential intervention, asymptomatic clients could attend a clinic without having to consult a health care provider. This had one of the highest prioritizations from both groups of experts, with 88% (38/43) of the CEs and 100% (39/39) of the PEs rating it as a priority at the end of their respective rounds (Figure 3). Express testing was excluded from round 3 of the PEs because it had reached unanimous consensus in round 2.

Similar to online-based testing, express testing was prioritized by most of the CEs and PEs. Both groups of experts identified that express testing would increase accessibility and availability and that it was quick, easy, and convenient. PEs reported having implemented this intervention at some clinics and that this option balanced the need for provider involvement against reducing time waiting to see a provider. However, some of the CEs worried that it did not decrease embarrassment or shame because the individual concerned could still be seen attending a clinic and that it could lead to more errors with self-collection, particularly anal swabs. PEs commented that they would need additional information on how testing using blood samples would be incorporated and how the contacts of a person testing positive for an STI would be managed. Some of the PEs had concerns that there were fewer opportunities to provide health education and counseling:

Express testing would reduce strain on sexual health clinics which is very helpful. However, it would depend on the detail of the collection instructions and the confidence that samples were collected correctly.

CE 022

This would be a high priority to the patients. It also is a priority for providers mostly because it would allow us to focus on patients who have symptoms or positive results. It does not however allow the opportunity for behavior counseling and other opportunistic interventions.

PE 019

### Nurse-Led Testing at Primary Care Clinics

**Figure 4:**
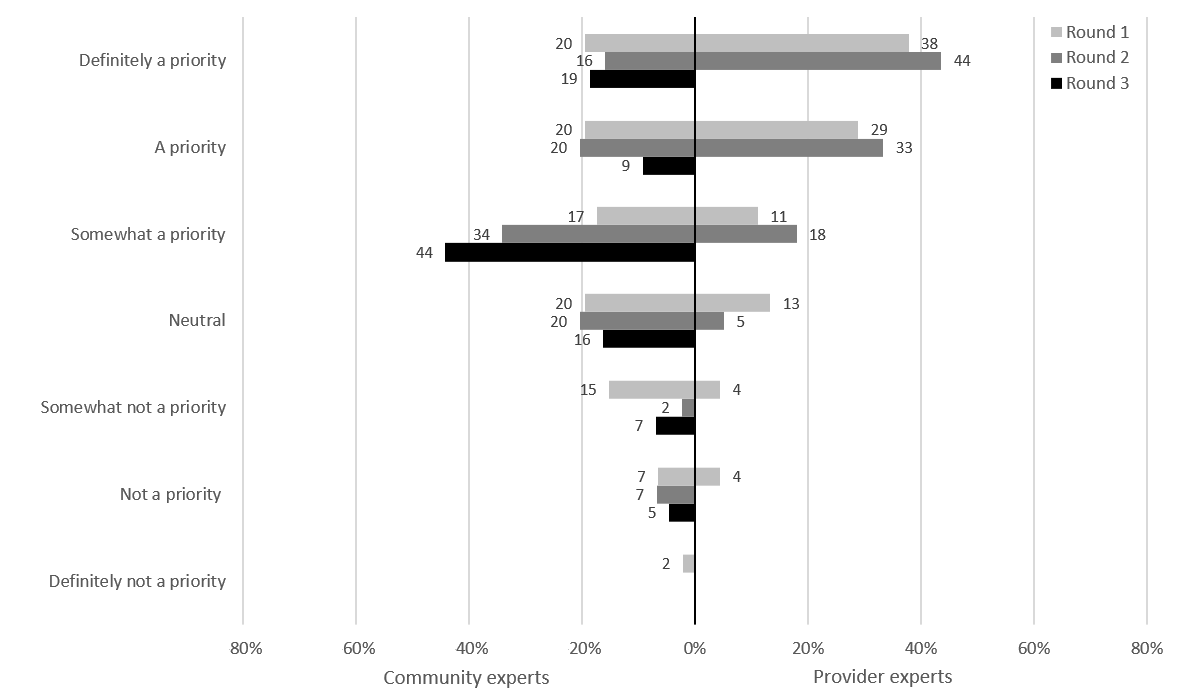
Nurse-led testing: prioritization by participants.

For this potential intervention, clients would be screened by a nurse who would then direct clients who are symptomatic to a physician, whereas asymptomatic clients would be offered testing based on sexual history. Of the CEs, 72% (31/43) rated nurse-led testing as a priority at the end of round 3 (Figure 4). Of the PEs, 95% (37/39) rated nurse-led testing as a priority at the end of round 2; as such, it was not presented in round 3 because of this nearly unanimous consensus.

PEs were enthusiastic about nurse-led testing because they saw it as cost-effective as well as more efficient, and patients could maintain a relationship with a provider. However, they needed additional information on who would pay and provide support for nurses, medical directives to assign responsibilities, and clarification on the role of counseling. Although this was already happening in some clinics, not all clinics have a nurse to whom this role can be assigned. CEs were agreeable to having nurse-led testing provided that the nurse was nonjudgmental, friendly, and knowledgeable about the gay and bisexual men’s community because a few singled out previous nurse encounters as stigmatizing:

Nurse-led testing is great, and I think essential because it allows you to ask questions that you may need opinions and resolutions for. If it was my first time being tested, I would probably prefer that option.

CE 014

This option currently exists at [my] clinic, but the nurses also have medical directives to treat STIs at the same visit based on a screening...It works well as the nurses also work under medical directives to provide counseling, treating, and health education. Not only does this allow the establishment of a therapeutic relationship, but it also enables patients to be more comfortable in accessing health care services in a timely manner.

PE 012

### Routine Testing

**Figure 5:**
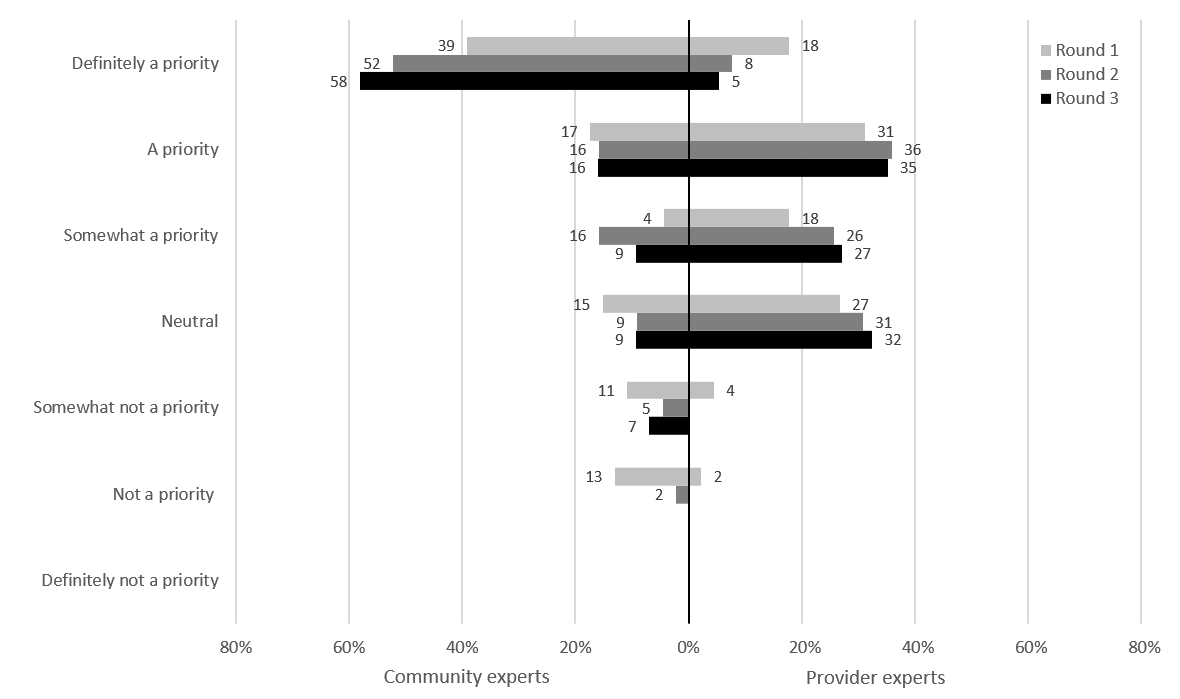
Routine testing: prioritization by participants.

Routine testing was described as standing orders at annual visits, regular care such as HIV care or PrEP visits, or visits not related to sexual health. Of the CEs, 84% (36/43) rated routine testing as a priority at the end of round 3 (Figure 5). Of the PEs, 68% (25/37) rated routine testing as a priority at round 3, but 32% (12/37) rated it as neutral.

Both CEs and PEs agreed that routine testing would help to normalize asymptomatic STI testing and that this was already happening for many GBM. However, it was emphasized that this was dependent on the relationship with the provider. If a client was out about their sexual orientation and comfortable discussing GBM issues with their provider, testing was straightforward and provided continuity of care. However, it was identified by both groups that GBM may not have a family physician or attend clinics frequently enough or they may not be practicing high-risk sexual behaviors. Logistics was another concern flagged by both groups because routine testing did not solve the issue of wait times or the need to prioritize other medical issues during an appointment. In addition, this could lead to overtesting and an inappropriate use of health care dollars:

Many people are busy and forget about all the testing they have done or needs to be done, I think it’s a good idea to have a calendar base routine testing at the Dr’s office to remind both doctors and patients about the testing.

CE 037

This would promote sexual health as an essential component of overall health, potentially decreasing stigma. People without primary care providers would not have access and primary care providers require more training on LGBTQ+ [lesbian, gay, bisexual, transgender, queer, and similar minority] and MSM [men who have sex with men] competence.

PE 050

### Client Reminders

**Figure 6:**
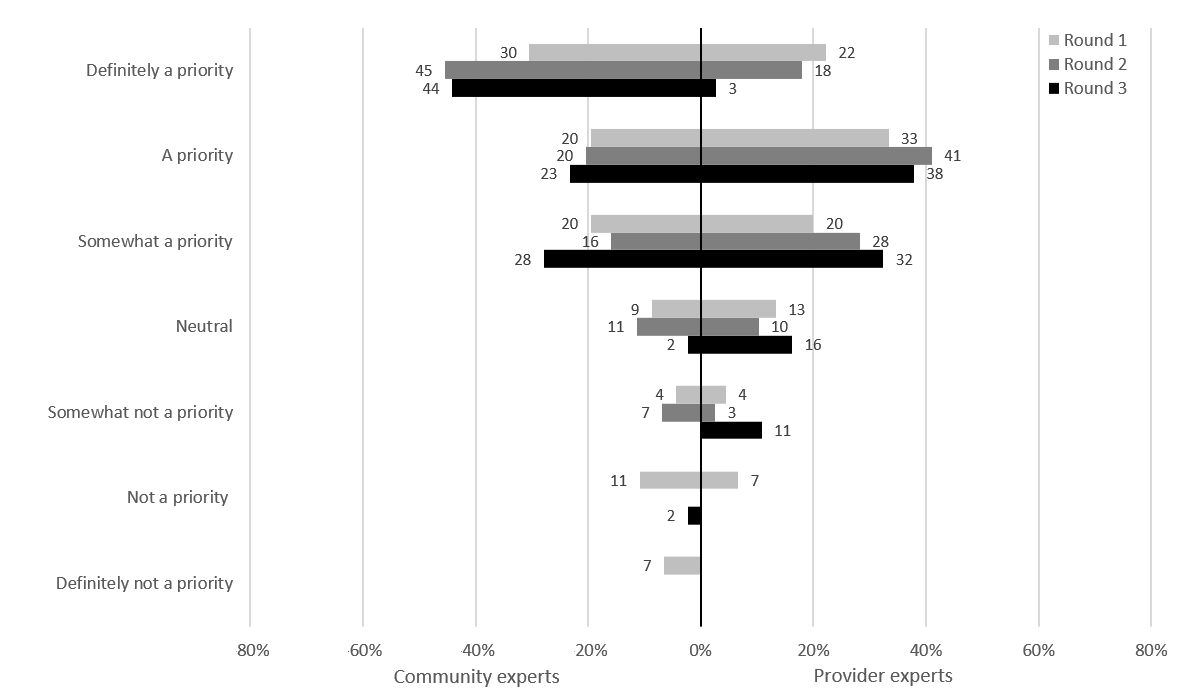
Client reminders: prioritization by participants.

For this intervention, clients would opt in to receive reminders by SMS text message, email, or mail. Of the CEs, 95% (41/43) rated client reminders as a priority at the end of round 3 (Figure 6). However, PEs rated client reminders as a lower priority (27/37, 73%) at the end of round 3, with 27% (10/37) rating it not a priority or choosing the neutral rating.

CEs rated client reminders highly because they routinized testing, particularly for those who forgot or did not think that they were at risk for STI. Some of the CEs felt that client reminders were convenient self-check reminders sent to their mobile phone, with many already using such a feature as part of their PrEP or HIV care. However, some of the CEs expressed concerns about privacy, whereas others felt that reminders were not needed because testing was not warranted or could be anxiety inducing. It was also flagged how easy it is to ignore such reminders:

I think it’s a good idea to have the reminders be sent out but it doesn’t say how often they would need to be tested? If someone needs a follow up or is high risk then there are specific intervals to come back to be tested, so the reminders would be a good idea. It would be even better if they could make an appointment. However, if there are no symptoms and at low risk, the patient doesn’t need reminders to come back. It would be up to the patient if they feel they need an appointment.

CE 005

PEs shared similar opinions, stating that it was beneficial for those who are busy to have automated reminders to normalize testing. Conversely, they identified that many clinics do not have the infrastructure to support reminders, and reminders had diminishing returns and would not help those not connected to care:

This would really further optimize those patients who are already utilizing the healthcare system to better engage and would overall I think make this experience more “hassle-free” as well as a more enjoyable, collaborative experience.

PE 058

### Online Booking App for STI Testing

**Figure 7:**
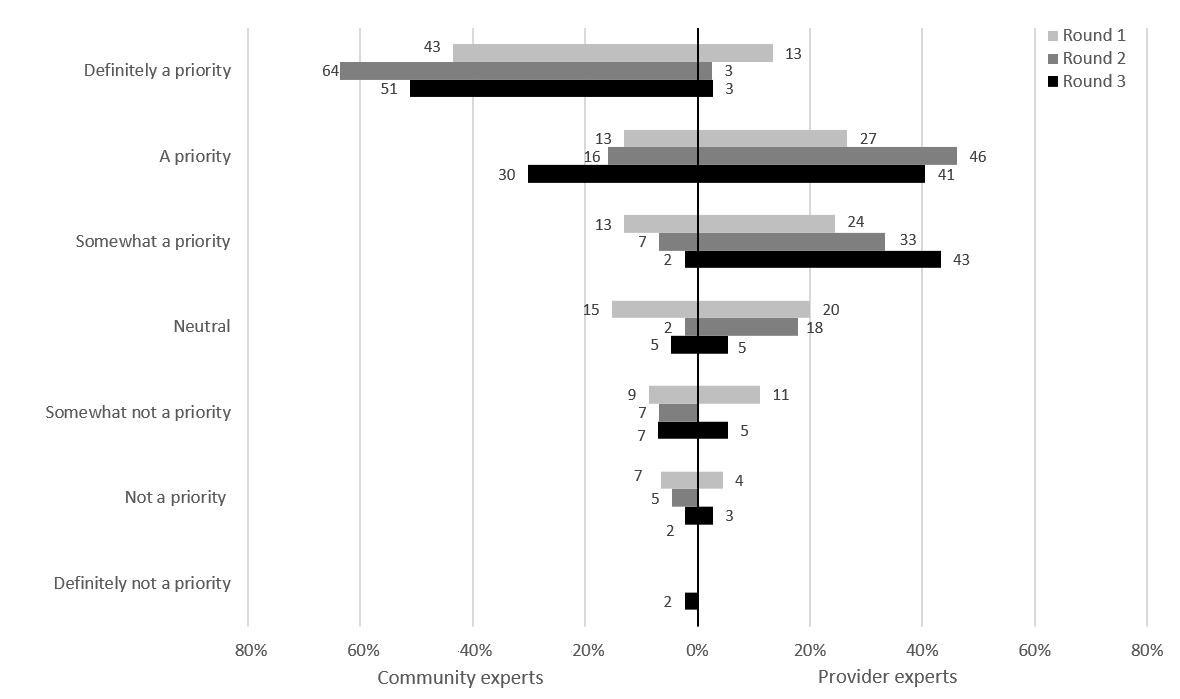
Online booking app: prioritization by participants.

This intervention was described as an online booking app or website to be used by clients searching for information on STIs and local clinics to get tested. Of the CEs, 84% (36/43) rated an online booking app as a priority at the end of round 3 (Figure 7). Of the PEs, 87% (32/37) rated an online booking app as a priority at the end of round 3.

Both PEs and CEs identified that an online booking app would be beneficial, efficient, and convenient. The experts commented that “we are already online and need to leverage technology,” and many of them preferred digital platforms provided that they are designed well. A booking app could increase accessibility and availability but only for those who had access to technology. In addition, this came with the disadvantages associated with clinics, including increased wait times, inconvenient opening hours, physical examinations, and potentially being judged. PEs asked how an online booking system would be integrated with existing clinic appointment systems and reported that a low-barrier booking system often led to a higher number of no-shows:

This is the best idea on the list. I think that having an app to find slots, book times, and perhaps see results is absolutely genius. It works well for busy people who need a convenient time slot over regularity at a given clinic. It catches those who don’t have primary care doctors. It would be a platform for the dissemination of information regarding safe sex, especially for how to have “kinky” sex in a safe way; this is an issue many doctors do not even know about...i.e., chemsex, slamming, fisting, group sex, and even bug chasers.

CE 031

Our clinic sometimes has a bottleneck in accessing reception by phone to book appointments. This would allow people to book without having to disclose a reason for visit and would free up reception staff to attend to other action items. One drawback is it requires people to be self-motivated to learn about risk—this would work for people who traditionally over-estimate or accurately estimate their risk but may not be deemed a priority by people who consistently underestimate their risk levels.

PE 014

Among CEs, minor differences were seen between White GBM and GBM reporting another ethnoracial identity for prioritizations at the end of round 3. Compared with White GBM, GBM who reported an identity other than White were more likely to prioritize online-based STI testing (a priority: 9/13, 38% vs 4/13, 21%) and the online booking app (definitely a priority: 14/22, 58% vs 8/22, 42%), commenting that they favored digital engagement when possible.

### Provider Alerts

**Figure 8:**
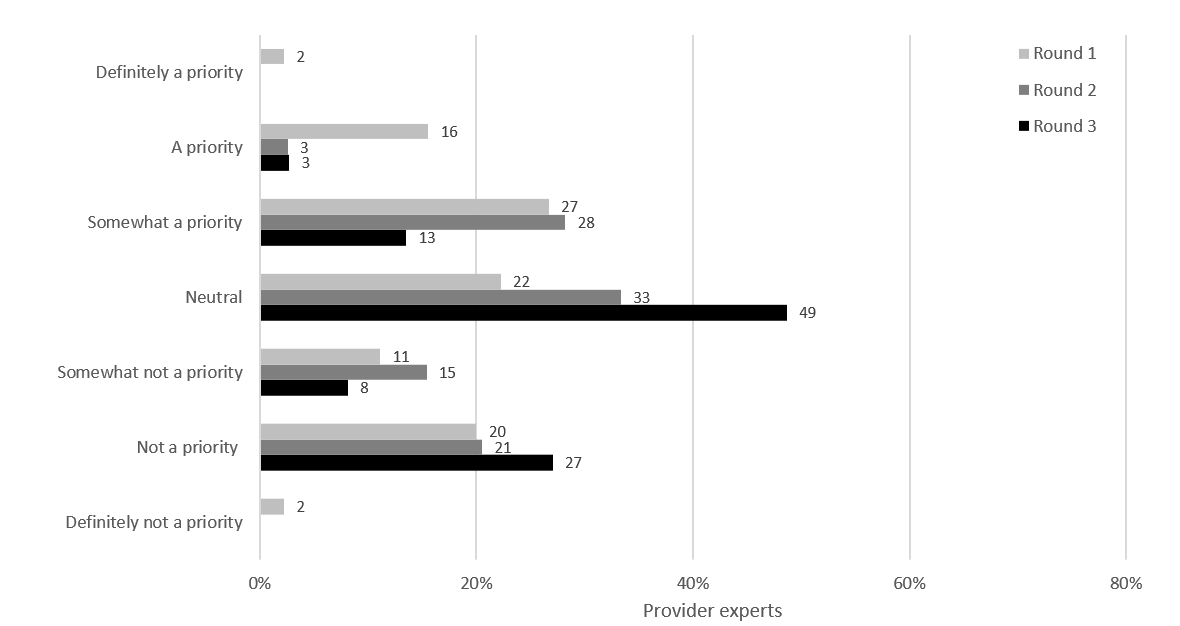
Provider alerts: prioritization by participants.

For the provider alerts intervention, shown only to PEs, providers would be prompted by the electronic medical record (EMR) system to offer STI testing based on a client’s self-reported sexual activities. By the end of round 3 of the survey, opinions varied widely, with no strong consensus reached (Figure 8). More than one-third (13/37, 35%) of the PEs said that it was not a priority, 49% (18/37) were neutral, and 16% (6/37) said that it was a priority.

Some of the PEs said that provider alerts had benefits because this intervention helped to make STI testing a part of routine care and could be useful for providers who provide sexual health care less often by decreasing stigma. However, other PEs had concerns because they are oversaturated with alerts or felt that the sexual history questionnaire would not adequately capture changes in risk behavior compared with taking a sexual history and providing counseling or preferred that the patient initiate testing. Additional information was wanted on how the sexual history questionnaire would be integrated into EMRs, particularly if they did not have a functioning EMR system:

As part of a multi-pronged approach this could be effective. Again, this would emphasize the importance of sexual health being considered a part of overall health, potentially decreasing stigma. Since there is no universal EMR record currently, it would be difficult to implement this on a large scale.

PE 030

We already have some reminders which are helpful but sometimes too many reminders, providers get oversaturated and start ignoring them. There is a fine balance—I also don’t see this reducing barriers for our most at-risk clients.

PE 020

### Provider Audit and Feedback

**Figure 9:**
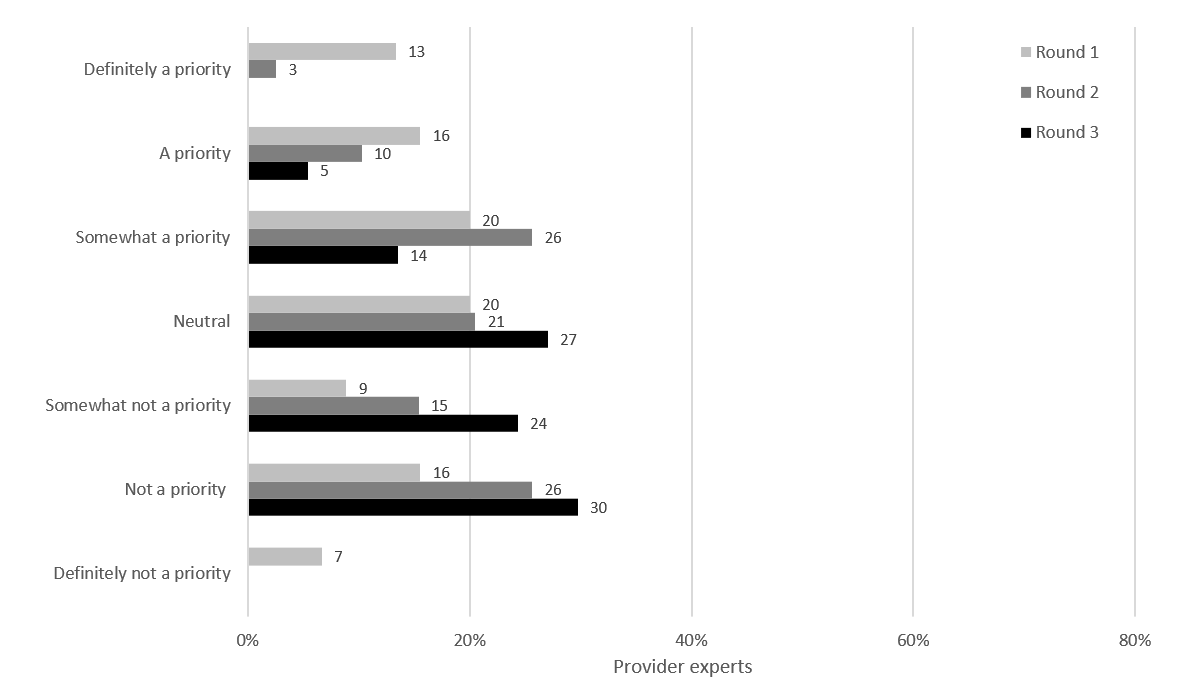
Provider audit and feedback: prioritization by participants.

Provider audit and feedback, shown only to PEs, was a potential intervention where providers would receive customized reports about their own STI testing practices and patterns with strategies to improve or reinforce good performance. At the end of round 1, opinions on provider audit and feedback were evenly distributed, with 49% (22/45) of the PEs identifying it as a priority (Figure 9). By the end of round 3, opinions shifted widely, with no strong consensus reached: 54% (20/37) said that it was not a priority, 27% (10/37) were neutral, and 19% (7/37) said that it was a priority.

Some of the PEs said that it was helpful to reflect on their practices as part of quality improvement, but they would require additional supportive infrastructure, such as physician education and training. Because of the time required and workload involved, as well as the administrative capacity needed to collect feedback, implement changes, and evaluate the outcome of these changes, many of the PEs felt unsure whether provider audit and feedback would result in a sustained increase in testing. Some of them thought that health care providers would not participate. Some of them thought that there would be difficulty in comparing different providers, practices, and populations. PEs commented that they would need additional information on how the feedback would be used to evaluate their practices and develop future goals for providers:

I think this is a priority because I believe quality improvement and practice management is important to assess and reassess throughout one’s career.

PE 037

As providers, we often have numerous screening targets and parameters to monitor. I'm not sure this would be a priority for everyone and would require a culture in each setting that identifies it as such, not just for the providers, but also the admin staff how would prepare the reports. Then providers would have to act on them. It seems other options would be as effective and more efficient.

PE 014

### Ranking the Top 3 Interventions

Participants also ranked their top 3 interventions at the end of round 2 from the interventions presented to them. They were subsequently shown the overall rankings and asked to rerank the top 3 at the end of round 3; if they selected an option that was not in the top 3, they were asked to explain why they had done so. For CEs, the top 3 options at the end of both rounds 2 and 3 were express testing, routine testing, and a booking app, although all 6 interventions were closely ranked. CEs who selected other options reported that it was because another intervention suited them based on their personal circumstances. For PEs, the top 3 options at the end of both rounds 2 and 3 were express testing, online-based testing, and nurse-led testing, with a clear divergence emerging between the top 3 and the remaining 5 interventions. PEs who selected an alternative to the top 3 felt that the clinic would still be the first point of contact or identified a lack of infrastructure and support for the top 3 options.

## Discussion

### Principal Findings

Our e-Delphi study in Toronto aimed to reach consensus among CEs and PEs for prioritizing interventions for implementation to increase bacterial STI testing among GBM. Both panels were enthusiastic about innovations that make STI testing more efficient, with express testing rating highly in both the prioritizations and top 3 rankings. However, CEs preferred convenient interventions that involved their provider, whereas PEs favored interventions that prioritized patient independence and reduced patient-provider time.

We reached consensus among CEs, for whom the top 3 interventions were express testing, routine testing, and a booking app. This suggests that GBM generally want more options to testing, rather than restricting it to 1 intervention. Overall, CEs emphasized that they wanted testing to be accessible and convenient while being able to maintain a relationship with a provider that was nonstigmatizing and nonjudgmental.

Among PEs, the top 3 interventions were express testing, online-based testing, and nurse-led testing, identified as a priority by 95% (37/39) to 100% (39/39) of the participants at the end of round 2. This indicates a desire to find opportunities away from the provider to ease the existing burden of testing. Initially, providers highly prioritized routine testing, client reminders, and a web-based app for booking testing, but reactions became more ambivalent by the end of round 3, although they still reached consensus identifying the intervention as a priority. We did not reach consensus among PEs for provider alerts or provider audit and feedback, with many expressing neutral or negative opinions about these options.

Similar themes that emerged from both groups of experts included shortened wait times and using technology to maximize convenience. Previous methods that incorporate these features range from computer-assisted self-interview [34] to web-based services such as GetCheckedOnline in British Columbia, Canada [35], which have been well received. Work by Brennan et al [36] shows that GBM are seeking STI information online, with 46% searching for information on HIV or STI testing, suggesting that GBM are open to using online methods for testing. Both CE and PE groups agreed on the need to normalize testing and the difficulties in normalizing testing, such as using client reminders that were easy to ignore or fearing a drain on resources with routine testing.

Conversely, the 2 groups differed on the role of the primary provider. Rapport and relationships with providers were highlighted by CEs, whereas PEs’ preferences tended toward decreasing the burden on existing services and task shifting. This echoes our previously reported findings from a provider survey where 40% of the providers at sexual health clinics and 65% of the primary care providers reported *insufficient consultation time* as a barrier to testing [30]. Conversely, CE preferences align with the findings from a scoping review by Peckham et al [37], where patients indicated that they desired personalized care that was respectful and culturally competent, holistic care that was nonjudgmental and coordinated continuous care with a primary care provider. The divergence in preferences highlights the different needs of, as well as barriers and facilitators experienced by, community members versus those of health care providers. A suite of diverse approaches may best allow individuals to access services that best suit their particular needs while optimizing the delivery of available health care resources.

The fact that rounds 2 and 3 of the PE e-Delphi survey took place after Toronto began its COVID-19 shutdown may have influenced our observation that >95% (>37/39) prioritized express testing, online-based testing, and nurse-led testing because they would minimize face-to-face provider-patient encounters. The highest number of PEs lost to follow-up occurred between rounds 1 and 2, possibly because of the redeployment of providers at public health units, whereas others adjusted to remote care and working from home environments. With this increase in client independence, questions arose around logistics, such as managing follow-up and integrating relevant information into existing EMR systems. PEs remarked upon the need for additional funding and support because many of the interventions had elements that were not accessible to them; for example, providers in primary care settings pointed out that they did not have nurses on staff, whereas others reported that their EMR system lacked the technological capability to automate reminders or alerts. These systems-level issues underscore acute concerns with current resource allocation and delivery of health care services.

### Strengths

The strengths of our Delphi study include high retention: 93% (43/46) of the CEs and 82% (37/45) of the PEs returned for the final round. We exceeded our CE diversity targets, with 52% (24/46) reporting an ethnoracial identity other than White, and 76% (35/46) were aged 18 to 39 years. The PEs were highly experienced in sexual health care, with 62% (28/45) ordering ≥20 bacterial STI tests per month. By conducting separate Delphi studies for community members and providers, we maximized willingness to share perspectives and experiences from both users and providers of STI testing services.

### Limitations

There are limitations to note. Experts for this study were self-identified as belonging to either community or provider group, and providers were identified through our professional networks, possibly limiting variability in perspectives. We purposively sampled community participants already engaged in seeking STI testing; as a result, their prioritizations may not be generalizable to community members who are not engaging in existing services. Our use of an anonymized online format allowed all participants to voice their opinions on the same level playing field; however, this would have excluded participants without access to the internet. We purposely limited our study to bacterial STIs and excluded HIV; the results may have differed had we asked about HIV testing. We observed some differences in prioritizations between White and non-White GBM at the end of round 3; however, our sample size was not sufficiently powered to detect statistically significant differences within the panels. We recommend that future studies examine differences in preferences according to ethnoracial identification. It is also unknown to what extent the COVID-19 pandemic influenced PEs’ prioritizations of the interventions. Future investigators may wish to expand the list of potential interventions to include more options, including remote care and telephone- or video-based consultations, which became widespread during the pandemic [38]. Unlike other Delphi studies, this study has not resulted in a clinical guideline or recommendation [27], but components could be adapted into current practice.

### Conclusions

The findings from this e-Delphi study point to areas of consensus regarding interventions with the greatest potential for improving local STI testing services for GBM communities in Toronto. Express testing was the intervention of choice among both CEs and PEs because it balanced efficiency with the preference to consult with a provider. CEs preferred convenient interventions that involved their provider, whereas PEs favored interventions that prioritized patient independence and reduced patient-provider time. No consensus was reached regarding provider alerts and provider audit and feedback among members of our PE panel, suggesting that efforts to introduce such interventions may benefit from advance preparatory investigations to identify and mitigate barriers. Altogether, our findings suggest that a reorganization of the current structure of sexual health services, rather than an entirely novel intervention, would best serve both community and provider preferences. Considering the changes forced upon health care delivery by the COVID-19 pandemic, this may be more achievable than previously thought with widespread adoption of remote care and reduced face-to-face appointments. The elements presented in these interventions could be implemented quickly and effectively to curb transmission of chlamydia, gonorrhea, and syphilis among GBM.

## Appendix

Multimedia Appendix 1 Description of interventions for participants.

## Abbreviations

CE: community expert
EMR: electronic medical record
GBM: gay, bisexual, and other men who have sex with men
PE: provider expert
PrEP: pre-exposure prophylaxis
STI: sexually transmitted infection

## Data Availability

The data sets generated and analyzed during this study are not publicly available because they contain information that could compromise the privacy of research participants. Data are available from the corresponding author (ANB) on reasonable request.
