# Supplementary material for: Community and Health Care Provider Preferences for Bacterial Sexually Transmitted Infection Testing Interventions for Gay, Bisexual, and Other Men Who Have Sex With Men: e-Delphi Study
Source: J Med Internet Res. 2023 Jun 29;25:e40477. doi: 10.2196/40477 (PMC10365575; doi:10.2196/40477)
Supplement: Multimedia Appendix 1 [file jmir_v25i1e40477_app1.docx]

# Supplementary material: Description of Interventions for Participants

## Interventions shown to Community Experts

### A. Online-based Bacterial STI Testing

For Online-based Bacterial STI testing, you would order your test from a website, download a lab form and visit a lab in person to provide samples (for example, anal swab, throat swab or urine samples for chlamydia and gonorrhea, or blood sample for syphilis).

What you would need to do:

· Answer questions about sexual history and signs and symptoms of STIs on a website

· Download a lab form and go to a lab to complete testing

· Might need to collect your own samples (for example anal swab, urine sample)

· Get test results online or by phone

· Go to a clinic or doctor’s office if you had symptoms or got a positive test result

What you would NOT need to do:

· Don’t need to get a physical exam

· Don’t need to go to a clinic or doctor’s office to get the test

### B. Express Testing at Clinics with Self-collection of Samples

You go to a clinic and fill out symptoms and sexual history questionnaire. If you have symptoms, you would see a doctor. If you have no symptoms, you proceed directly to collect your own samples for testing. To collect your own samples, you will be provided with a private space with posters describing how to collect each sample. Samples that could be self-collected include: urine, rectal swab or throat swab. The collected samples are placed in a bin and you leave the clinic. Self-collected samples would include testing for chlamydia and gonorrhea.

What you would need to do:

· Visit to a clinic

· May require an appointment

· Collect your own samples

What you would NOT need to do:

· Don’t need to get a physical exam

· Don’t need to spend a lot of time spent in clinic

· Don’t need to talk about your sex life with a health care provider

C. Nurse-led Testing in Primary Care Clinics
You go to your family doctor office and are seen by a nurse who asks questions about STI symptoms and sexual history. If you have symptoms, you will see the doctor. If you do not have symptoms, your sexual history helps determine which tests are needed. You would be called back if the results of the test are positive.

What you would need to do:

·         Visit to a clinic

·         May require an appointment

·         Share information with nurse about your sexual life

·         Provide samples, which may be collected by the nurse or self-collected

What you would NOT need to do:

·         Don’t need to spend a lot of time in clinic

·         Don’t need to get a physical exam   

D. Routine Testing 
You visit your doctor for any reason and STI testing is ordered automatically. This might be at your annual doctor visit, as a part of HIV care (if you are living with HIV), or when you see your doctor for another health issue, such as strep throat or pain in your shoulder.  
    
 What you would need to do:

·         Visit a clinic for other reasons

·         Spend some time in a waiting room

·         Provide samples, which may be collected by the doctor or self-collected

What you would NOT need to do:

·         Don’t need to talk about your sex life to get tested

E. Client Reminders 
You see a healthcare provider for bacterial STI testing and give permission to receive reminders in the future. When it is time for your next bacterial STI test, you would receive the reminder as a text message (SMS), email or mailed letter.
The text would look something like this: 
"Hi (Patient name) It’s time for a routine test. Walk-in, call xxxxxx or email xxxxxxxx for an appointment"

What you would need to do:

·         Need to be able to receive notifications when it's time to go in for another test by text message (SMS), email, or mailed letter

·         Visit a clinic

·         Spend some time in a waiting room

·         Provide samples, which may be collected by the provider or self-collected   
 
What you would NOT need to do:
·         Don't have to remember when it's time to go in for another test

F. Online App for Booking STI Testing    
You use an app or visit a website that has information about bacterial STIs, such as the ways that these infections can be transmitted, the symptoms of infection, and how the tests are done. You can use the website/app to book an appointment for an STI test at a clinic near you.

What you would need to do:

·        Use the app to learn more about bacterial STIs and when to seek care

·         Use the app to book an appointment

·         Visit a clinic

·         May spend some time in a waiting room

·         Share information about your sexual life

·         Provide samples, which may be collected by the provider or self-collected   

What you would NOT need to do: 
·         Don't need to go anywhere else to make an appointment

## Interventions shown to Provider Experts

### A. Online-based Bacterial STI Testing

For Online-based Bacterial STI testing, the patient would request the test from a website, download a lab form and visit a lab in person to provide samples (for example, anal swab, throat swab or urine samples for chlamydia and gonorrhea, or blood sample for syphilis).

What would need to be done:

· Patient answers questions about sexual history and signs and symptoms of STIs on a website

· Patient downloads a lab form and goes to a lab to complete testing

· Patient may need to self-collect samples (for example anal swab, urine sample)

· Patient receives notification that test results are available online or by phone

· Patient visits a clinic if they have symptoms or a positive test result and are seen by healthcare provider

What would NOT need to be done:

· Patient does not need to get a physical exam

· Patient does not need to go to a clinic or doctor’s office to get the test

· Healthcare provider does not provide risk behavior counseling or offer care for other conditions if negative

### B. Express Testing at Clinics with Self-collection of Samples

Patient goes to a clinic and fills out symptoms and sexual history questionnaire. If symptomatic, the patient sees a healthcare provider and proceeds with a clinical exam. If asymptomatic, the questionnaire recommends which samples should be self-collected for testing. To self-collect samples, patients will be provided with a private space with posters describing how to collect each sample. Samples that could be self-collected include: urine, rectal swab or throat swab. Self-collected samples are placed in a bin for processing. There is no clinical exam.

What would need to be done:

· Patient may require an appointment

· Patient visits a clinic

· Patient answer questions about sexual history and signs and symptoms of STIs

· If patient is asymptomatic, they proceed to self-collect samples for testing

· Patient sees a healthcare provider if they have symptoms or a positive test result

· Space and materials are needed for self-collection of samples

What would NOT need to be done:

· Patient does not need to get a physical exam

· Patient does not need to spend a lot of time in clinic

· Patient does not need to talk about their sexual activities with a healthcare provider

· Healthcare provider does not need to see asymptomatic patients

· Healthcare provider does not provide risk behavior counselling or offer care for other conditions if negative

### C. Nurse-led Testing in Primary Care Clinics

Patient attends a primary care clinic and is screened by a nurse or allied health professional who asks questions about STI symptoms and sexual history using standardized questions. If the patient has symptoms, they will see the doctor. If the patient does not have symptoms, their sexual history helps determine which tests should be offered. Patients are called back if the results of the test are positive and treatment is required.

What would need to be done:

· Patient may require an appointment

· Patient visits a primary care clinic

· Patient shares information with nurse or allied health professional about their sexual activities

· Patient provides samples, which may be collected by the nurse or allied health professional or self-collected

· Patient sees a healthcare provider if they have symptoms or a positive test result

· Need to add staff to the team and have the physical space to be able to see more clients in clinic

What would NOT need to be done:

· Patient does not need to get a physical exam

· Patient does not need to spend a lot of time in clinic

### D. Routine Testing

STI testing is routinized using standing orders - tests that are provided at healthcare visits for other

purposes, such as annual visits and as a part of HIV care (if they are living with HIV), or when they see their doctor for another health issue, such as strep throat or pain in their shoulder.

What would need to be done:

· Patient visits a clinic for other reasons

· Patient spends some time in a waiting room

· Patient provides samples, which may be collected by the doctor or self-collected for testing

· Normalizes testing and minimizes stigma associated with STI testing

· Patient tested may have minimal risk

What would NOT need to be done:

· Patient does not need to talk about their sexual activities to get tested

### Client Reminders

Patients who have had STI tests before and have consented to being contacted between visits would receive reminders to return for their next STI test via SMS text, email or mail.

The reminder message would look something like this:

"Hi (Patient name), it’s time for a routine test. Walk-in, call xxxxxx or email xxxxxxxx for an appointment"

What would need to be done:

· Patients need to visit a clinic and engage in the healthcare system

· Patients need to be able to receive notifications when it's time to come in for another test by text message (SMS), email, or mailed letter

· Requires institution privacy approval and patient consent to receive communication between visits

· Requires infrastructure to send reminders automatically

What would NOT need to be done:

· Patient does not have to remember when it's time to come in for another test

· Reminders to patients do not use many resources if reminder system is automated

### F. Online App for Booking STI Testing

Patient uses an app or visits a website that has information about bacterial STIs, such as the ways that these infections can be transmitted, the symptoms of infection, and how the tests are done. Patient can use the website/app to book an appointment for an STI test at a clinic near them.

What would need to be done:

· Patient uses the website/app to learn more about bacterial STIs and when to seek care

· Patient uses the website/app to book an appointment

· Patient visits a clinic

· Patient may spend some time in a waiting room

· Patient shares information about their sexual activities with healthcare provider

· Patient provides samples, which may be collected by a healthcare provider or self-collected

· Booking services need to be tailored to specific clinic policies and procedures

· Requires development and maintenance of infrastructure for online application and booking services

What would NOT need to be done:

· Patient does not need to go anywhere else to make an appointment

### G. Provider Alerts

Healthcare providers receive alerts through electronic medical records (EMR) systems to prompt an offer of STI testing. Alerts could be triggered when clients are due for STI re-testing or if a client self-completes a sexual history questionnaire and reports high-risk sexual activity.

What would need to be done:

· Patient makes an appointment and visits a clinic

· Patient spends some time in a waiting room

· Patients answers a self-completed sexual history questionnaire

· Modify EMR system or require additional technology for customized alerts based on answers from patient’s self-completed history questionnaire

· Providers must notice the alert and act on it

· Patient provides appropriate samples, which may be collected by the healthcare provider or self-collected

What would NOT need to be done:

· Healthcare provider does not need to take a sexual history

· Healthcare provider does not need to review chart to see if patients need an STI test to offer the test as responses from self-completed sexual history questionnaires would trigger alert

· Healthcare provider does not need to remember to offer STI testing to patients who would benefit from testing

### H. Provider Audit and Feedback

A person or organization has access to healthcare provider-level STI testing data and prepares regular reports. Healthcare providers receive regular customized reports about their own STI testing practice patterns, and how it compares to guideline recommendations, their own historical STI testing pattern, and other providers. The report also contains actionable goals to achieve STI testing targets, encouraging improvement and reinforcing good performance. It also promotes healthy competition by showing providers how they are doing relative to their peers.

What would need to be done:

· Healthcare providers have to commit time to review and reflect on their performance and make changes to their practice, if appropriate

What would NOT need to be done:

· Healthcare providers do not need to complete a detailed chart review to seek out own testing data
